# Effects of feeding a thermomechanical, enzyme-facilitated, coprocessed yeast and soybean meal on growth performance, organ weights, leg health, and gut development of broiler chickens

**DOI:** 10.1016/j.psj.2023.102578

**Published:** 2023-02-11

**Authors:** Elena Colombino, Mark Karimi, Mai Anh Ton Nu, Andrea Aurora Tilatti, Sara Bellezza Oddon, Franco Calini, Cinzia Bergamino, Edoardo Fiorilla, Marta Gariglio, Francesco Gai, Maria Teresa Capucchio, Achille Schiavone, Laura Gasco, Ilaria Biasato

**Affiliations:** ⁎ Department of Veterinary Sciences, University of Turin, Grugliasco 10095, Italy; † AB Neo a/s, Videbaek, Denmark; ‡ Department of Agricultural, Forestry and Food Sciences, University of Turin, Grugliasco, Italy; § Tecnas, Lugo, Italy; # Institute of Science of Food Production, National Research Council, Turin, Italy

**Keywords:** broiler chicken, poultry, coprocessed yeast and soybean meal, gut health, growth performance

## Abstract

The development of a healthy gut during prestarter and starter phases is crucial to drive chicken's productivity. This study aimed to evaluate the effects of a thermomechanical, enzyme-facilitated, coprocessed yeast and soybean meal (pYSM) on growth performance, organ weights, leg health, and gut development in broiler chickens. A total of 576 as-hatched broiler chicks were randomly allotted to 3 dietary treatments (8 replicates/treatment, 24 chickens/replicate): a control group (**C**) without the pYSM, a treatment group 1 (**T1**), in which the pSYM was included at 20, 10, 5, 0, and 0% levels in the prestarter, starter, grower, finisher I, and finisher II feeding phases, respectively, and a treatment group 2 (**T2**), in which the pSYM was included at 5, 5, 5, 0, and 0% levels in each feeding phase. On d 3 and 10, 16 broilers/treatment were euthanized. The T1 broilers tended to show higher live weight (d 3 and 7) and average daily gain (prestarter and starter phases) than the other groups (*P* ≤ 0.10). Differently, pYSM-based diets did not influence the growth performance of the other feeding phases and the whole experimental period (*P* > 0.05). Relative weights of pancreas and liver were also unaffected by pYSM utilization (*P* > 0.05). Litter quality tended to have higher average scores in C group (*P* = 0.079), but no differences were observed for leg health (*P* > 0.05). Histomorphometry of gut, liver, and bursa of Fabricius was not affected by diet (*P* > 0.05). Gut immunity was driven to an anti-inflammatory pattern, with the reduction of IL-2, INF-γ, and TNF-α in the duodenum of treated birds (d 3, *P* < 0.05). Also, MUC-2 was greater in the duodenum of C and T2 group when compared to T1 (d 3, *P* = 0.016). Finally, T1-fed chickens displayed greater aminopeptidase activity in the duodenum (d 3 and 10, *P* < 0.05) and jejunum (d 3, *P* < 0.05). Feeding high levels of pYSM (10–20%) to broilers in the first 10 d tended to improve growth performance in the prestarter and starter phases. It also positively downregulated proinflammatory cytokines during the first 3 d, as well as stimulated the aminopeptidase activity in the prestarter and starter periods.

## INTRODUCTION

As the growing period of broiler chickens continues to shorten, the first days (0–10) after hatching have become increasingly important to ensure the maximal growth potential of chickens (Cheled-Shoval et al., 2011). In particular, this period represents 20 to 25% of the total production cycle, being the most challenging period in a chicken's life due to the adaptation from the in ovo to the external environment (Ravindran et al., 2021). Indeed, they have to switch to aerial breathing, to start thermal regulation, and to change from yolk lipid nutrition to oral nutrition with complex dietary constituents (Duan et al., 2021). This latter aspect is strictly related to the development of the gastrointestinal tract, which affects the broiler growth and health throughout its whole life (Dai et al., 2020; Ravindran et al., 2021). Furthermore, as the starter period represents a key stage to obtain good performance results at the end of the production cycle, and chicks also eat less when compared to the other phases of the production cycle, the farmer is more willing to invest in this phase.

Gut not only plays a key role in digestion and absorption of nutrients, but it also represents the first line of defense between the host and the luminal environment, protecting the chicks from exogenous pathogens (Proszkowiec-Weglarz et al., 2020). To perform these functions, the gut undergoes abrupt macroscopical and microscopical changes in both the prestarter and the starter phases. First, its relative weight and size increase rapidly to digest and absorb nutrients to meet the needs of the growing chicks (Dibner and Richards, 2004). Second, the increase in villus height, crypt depth, and submucosal thickness between d 4 and 10, as well as the higher release of pancreatic (trypsin, chymotrypsin) and brush border enzymes (maltase, sucrase, aminopeptidase), also contribute to the cleavage and uptake of nutrients (Uni et al., 1995; Ravindran et al., 2021). Third, the sterile gut is immediately colonized by a varied microbial community via the feed and environment, thus contributing to the maturation of the immune response, especially the gut-associated immunity (Duan et al., 2021). Therefore, any improvement in early gut maturation, digestive functions, and gut health shows a positive impact on the chicken's production performance (Cheled-Shoval et al., 2011).

To date, different early nutrition strategies have been proposed in chickens as valuable tools to drive gut health and development (Yegani and Korver, 2008). They mainly focused on offering high-quality and highly digestible protein and energy sources to young chicks, considering their specific needs and the immaturity of their gastrointestinal tract (Sujatha et al., 2017). Soybean meal (**SBM**) is the most used protein source worldwide, but—like most of the plant proteins—it has a high concentration of antinutritional factors (**ANFs**), which decrease its nutritive value (Beski et al., 2015). In order to increase the quality and availability of SBM for chicks, several processing strategies applied in single—such as heat treatment, extensive protein extraction and isolation, thermomechanical processing, fermentation, and enzymatic treatments—have been proposed to reduce ANFs and, in turn, improve digestibility and nutritional profile of SBM (Batal and Parsons, 2003; Jahanian and Rasouli, 2016; Kim et al., 2016; Rasmussen et al., 2021). Previous studies have shown that the partial replacement of SBM with heat-treated, fermented, concentrated, or isolated soy proteins in a starter diet may result in an improvement in body weight, feed intake, feed efficiency, and mortality rates of broiler chickens (Beski et al., 2015; Kim et al., 2016; Nabizadeh, 2018), as well as in an increase in apparent metabolizable energy (**AMEn**) and amino acids (**AA**) digestibility (Batal and Parsons, 2003). However, the combination of different processing strategies (such as thermomechanical and enzyme treatments) has rarely been tested (Marsman et al., 1997). In parallel, the supplementation of yeasts—whose cell walls are mainly composed of functional ingredients (such as mannan oligosaccharides [**MOS**] and β-glucans) (Faustino et al., 2021)—in diets for broiler chickens has recently been proven to be able to ameliorate the bird growth performance and their gut morphology, integrity, and immune response (Kim et al., 2022). However, the coprocessing effect of SBM and yeasts on birds’ performance and gut health parameters have not been investigated yet.

Therefore, the aim of the present study was to evaluate the effects of a thermomechanical, enzyme-facilitated, coprocessed yeast and soybean meal (**pYSM**) on growth performance, organ weights, leg health, histopathological findings, and gut development (by means of histomorphology, enzymatic, and gene expression analyses) in broiler chickens.

## MATERIALS AND METHODS

### Animals and Husbandry

The experimental trial was performed at the poultry facility of the Department of Agricultural, Forest and Food Sciences of the University of Turin (Italy). The experimental protocol was designed according to the guidelines of the current European Directive (2010/63/EU) on the care and protection of animals and approved by the Ethical Committee of the University of Turin (Italy) (Prot n. 0284800). A total of 576 as-hatched broiler chicks (Ross 308) from the same fairly young parent stock were randomly allotted to 24 pens. Each pen (1.20 m wide × 2.50 m long) was equipped with a feeder and a drinker, and rice hull as litter. The poultry house was equipped with a waterproof floor and walls, completely covered by tiles, and provided with an automatic ventilation system. During the first 3 wk, birds were heated by infrared lamps to maintain a suitable temperature, according to the standard breeding practices (Aviagen and Ross Broiler Management Handbook, 2018). The lighting schedule was 23 h light:1 h darkness until d 7 and then 18 h light:6 h darkness was adopted until the slaughtering age. At hatching (directly in the hatchery), all the chicks received subcutaneous vaccination against Newcastle disease and Gumboro disease, ocular vaccination against infectious bronchitis, and spray vaccination against coccidiosis.

### Coprocessing of Yeast and Soybean Meal

The pYSM (Alphasoy Gold, AB Neo a/s, Videbaek, Denmark) used in the present study is produced by coprocessing of high-protein, thermomechanical and enzyme-facilitated SBM, and selected yeast fractions (minimum 10,000 mg/g β-1,3/1,6 glucans and 5,000 mg/g MOS in the final product). Briefly, the coprocessing included 4 steps as follows: 1) the raw material SBM was first ground by a hammer mill to 500 µm average particle size; 2) subsequently, the material was mixed with yeast fractions and be conditioned using water and steam including an enzyme mixture as a processing aid; 3) this mixture went through a high temperature, high pressure process for a short time into extrudates and then drying and cooling to stabilize the product; and 4) the extrudates were milled by a hammer mill to 400 µm average particle size. The pYSM (whose proximate composition, macrominerals content, and AA profile is reported in Table 1) has been designed to support the healthy development of the gastrointestinal system and optimum microbiota balance during the early growth stage of the young animals by increasing the AMEn, nutrient digestibility, and protein digestion kinetics, and reducing the ANFs in SBM, and exploiting the prebiotic effects of yeast fractions.

**Table 1:** Nutrient content and digestibility of the coprocessed yeast and soybean meal.

Item^1^	
Proximate composition	% as is
DM	95.00
CP	52.00
EE	2.50
CF	3.70
Ash	6.70
AMEn, MJ/Kg	11.00
Macrominerals	g/kg as is
Calcium	3.00
Phosphorus	6.00
Sodium	0.40
Potassium	21.00
Chloride	0.10
Magnesium	3.70
Aminoacids	% as is (true digestible levels)
Lysine	3.10 (2.90)
Methionine	0.70 (0.60)
Cystine	0.70 (0.60)
Threonine	2.00 (1.80)
Tryptophan	0.70 (0.60)
Isoleucine	2.50 (2.30)
Leucine	4.10 (3.80)
Valine	2.50 (2.30)
Phenylalanine	2.70 (2.50)
Histidine	1.40 (1.30)
Arginine	3.70 (3.50)
Glutamic acid	9.20 (8.40)
Tyrosine	1.80 (1.60)

1 Abbreviations: AMEn, apparent metabolizable energy; CF, crude fiber; CP, crude protein; DM, dry matter; EE, ether extract.

### Diets

Three different isonitrogenous, isolipidic, and isoenergetic dietary treatments were considered, according to a 1 × 3 factorial arrangement (8 replicates/diet, 24 birds/pen): a commercial feed without the inclusion of pYSM (control diet: C), and 2 commercial feeds with the pYSM inclusion (treated diets: T1 and T2). For each dietary treatment, the diets were divided into 5 feeding phases: prestarter (d 0–3, crumbled feed), starter (d 4–10, crumbled feed), grower (d 11–21, pelleted feed), finisher I (d 22–35, pelleted feed), and finisher II (d 36–42, pelleted feed). The treated diets included 20, 10, 5, 0, and 0% (T1) or 5, 5, 5, 0, and 0% (T2) of the pYSM in the prestarter, starter, grower, finisher I, and finisher II feeding phases, respectively. A core feed including the same levels of the main feed ingredients (excluding coccidiostats, extruded soybean, L-valine, and pYSM) was prepared and used at a constant percentage (70%) in all the diets (Table 2). All the diets (Table 3) were formulated to meet or exceed the Aviagen broiler nutrition specifications (Aviagen, 2019). Feed and water were provided ad libitum.

**Table 2:** Diet composition and nutrient content of the core feed.

Item^1^	
Diet composition	% as is
Corn meal (7.5% CP)	52.22
Wheat meal (11.5% CP)	28.57
Soybean meal (47.5% CP)	13.10
Soybean oil	2.92
Calcium carbonate	0.69
Dicalcium phosphate	0.53
Sodium chloride	0.30
Sodium formate	0.14
DL-Methionine	0.31
Lysine HCL	0.30
L-Threonine	0.07
Choline	0.14
Phytase-xylanase mix	0.14
Mineral-vitamin premix^2^	0.57
Nutrient content	% as is
DM	87.29
CP	13.57
EE	4.55
ADF	3.80
NDF	10.19
Ash	3.81
Sodium	0.18
Lysine	0.82
Methionine	0.53
AMEn (MJ/kg)	13.62

1 Abbreviations: ADF, acid detergent fiber; CP, crude protein; DM, dry matter; EE, ether extract; GE, gross energy; NDF, neutral detergent fiber.

2 Mineral-vitamin premix: vitamin A (retinyl acetate), 12,500 IU; vitamin D3 (cholecalciferol), 3,500 IU; vitamin E (DL-a-tocopheryl acetate), 40 mg; vitamin K (menadione sodium bisulfide), 2.0 mg biotin, 0.20 mg; thiamine, 2.0 mg; riboflavin, 6.0 mg; pantothenate, 15.21 mg; niacin, 40.0 mg; choline, 750.0 mg pyridoxine, 4.0 mg; folic acid, 0.75 mg; vitamin B12, 0.03 mg; Mn, 70 mg; Zn, 62.15 mg; Fe, 50.0 mg; Cu, 7.0 mg; I, 0.25 mg; Se, 0.25 mg.

**Table 3:** Diet composition and nutrient content of the experimental diets.

	Prestarter (0–3 d)			Starter (4–10 d)			Grower (11–21 d)			Finisher I (22–35 d)	Finisher II (36–42 d)
Items^1^	C	T1	T2	C	T1	T2	C	T1	T2	C-T1-T2	C-T1-T2
Diet composition, % as is											
Core feed	70.00	70.00	70.00	70.00	70.00	70.00	70.00	70.00	70.00	70.00	70.00
Maxiban^2^	0.06	0.06	0.06	0.06	0.06	0.06	0.06	0.06	0.06	0.00	0.00
Monteban^3^	0.00	0.00	0.00	0.00	0.00	0.00	0.00	0.00	0.00	0.07	0.00
Lysine HCl	0.04	0.06	0.04	0.04	0.05	0.04	0.02	0.03	0.03	0.01	0.00
L-Threonine	0.05	0.05	0.05	0.05	0.05	0.05	0.03	0.03	0.03	0.01	0.00
DL-Methionine	0.13	0.13	0.13	0.13	0.13	0.13	0.07	0.07	0.07	0.04	0.00
Valine	0.02	0.01	0.02	0.02	0.01	0.02	0.01	0.01	0.01	0.01	0.00
Corn meal (7.5% CP)	0.00	2.92	0.72	0.00	1.47	0.72	5.88	6.59	6.59	8.66	10.53
Calcium carbonate	0.07	0.07	0.08	0.07	0.07	0.08	0.00	0.00	0.00	0.02	0.01
Dicalcium phosphate	0.70	0.72	0.71	0.70	0.71	0.71	0.73	0.73	0.73	0.11	0.00
Soybean oil	1.28	0.00	0.96	1.28	0.64	0.96	1.26	0.95	0.95	1.49	1.99
Soybean meal (47.5% CP)	27.65	5.98	22.23	27.65	16.81	22.23	16.94	11.53	11.53	9.58	2.47
Extruded soybean (36% CP)	0.00	0.00	0.00	0.00	0.00	0.00	5.00	5.00	5.00	10.00	15.00
pYSM^4^	0.00	20.00	5.00	0.00	10.00	5.00	0.00	5.00	5.00	0.00	0.00
Proximate composition, % as is											
DM	89.05	89.07	89.04	88.66	89.72	89.71	89.20	89.44	89.34	89.32	90.50
CP	23.59	23.67	23.75	23.46	23.07	23.25	21.19	21.19	21.07	18.12	17.98
EE	5.21	4.96	4.87	4.91	4.57	4.93	6.05	5.94	6.08	8.48	8.61
ADF	3.44	3.35	3.72	3.40	3.24	3.21	3.23	3.08	3.14	3.31	3.08
NDF	7.10	6.86	6.77	7.28	6.87	7.14	7.37	7.18	7.65	7.84	7.69
Ash	4.85	4.67	4.87	4.65	4.88	4.85	4.61	4.56	4.53	4.27	4.10
AMEn (MJ/kg)	12.76	12.76	12.76	12.76	12.76	12.76	13.18	13.18	13.18	13.60	14.02

1 Abbreviations: ADF, acid detergent fiber; AMEn, apparent metabolizable energy; C, control; CP, crude protein; DM, dry matter; EE, ether extract; NDF, neutral detergent fiber; pYSM, coprocessed yeast and soybean meal; T1, 20% inclusion of coprocessed yeast and soybean meal in the prestarter phase, 10% in the starter phase and 5% in the grower phase; T2, 5% inclusion of coprocessed yeast and soybean meal in the prestarter, starter, and grower phases.

2 Coccidiostat used in the prestarter, starter, and grower feeding phases.

3 Coccidiostat used in the finisher feeding phases.

4 Coprocessed yeast and soybean meal = a protein-rich product obtained from coprocessing of soybean meal and yeast fractions—Alphasoy Gold (AB Neo a/s, Videbaek, Denmark).

### Chemical Analyses of Experimental Diets

The concentrate nucleus and the diets were ground to pass through a 0.5-mm sieve and stored in airtight plastic containers. Dry matter (**DM**, method number 943.01), ash (method number 924.05), crude protein (**CP**, method number 954.01), ether extract (**EE**, method number 920.39), neutral detergent fiber (**NDF**, method number 2002.04), and acid detergent fiber (**ADF**, method number 973.18) were determined (AOAC International Arlington, 2019). The gross energy (**GE**) content was determined using an adiabatic bomb calorimeter (C7000; IKA, Staufen, Germany). All the analyses were performed in duplicate and expressed as average values (Tables 1 and 2).

### Growth Performance

The experimental trial lasted 42 d. Health status and mortality were daily monitored during the whole experimental period. The live weight (**LW**) was recorded at an individual level on d 3, 7, 10, 14, 21, 28, 35, and 42. The average daily gain (**ADG**), the daily feed intake (**DFI**) and the feed conversion ratio (**FCR**) were determined at the pen level for each feeding phase (d 0–3, 4–10, 11–21, 22–35, and 36–42) and for the overall experimental period (d 0–42). All the measurements were made using a high precision electronic scale (KERN PLE-N v. 2.2; KERN &amp, Sohn GmbH, Balingen-Frommern, Germany, d: 0.1).

The LW variations within the C- and the pYSM-fed groups were also evaluated at d 0, 14, 28, and 42 as follows:

- Coefficient of variation (**CV**, %), which measures the spread of LW within the groups and is calculated as the standard deviation (σ) divided by the mean LW weight of the group.
- Uniformity (%), which measure the evenness of LW within the groups and is expressed as the percentage of the group whose LW is ±10% of the mean LW of the group.

### Litter Quality and Feet and Hock Health Assessment

The litter quality was assessed always by the same operator through the “Pens Average Litter Scoring (**PALS**)” system, which is based on the percentage of “unacceptable” litter identified within each pen in different stages of the birds’ life. In particular, an “unacceptable” litter is wet or capped and unsuitable for chicken growing, while an “acceptable” litter is dry, slightly capped but friable. At the end of each feeding phase, the “unacceptable level” of the litter status was scored from 0 to 100 for each pen, being 100 the worse (100% unacceptable) and 0 the best (0% unacceptable).

The feet and hocks of the broiler chickens were examined on d 35 and 42 in order to assess the incidence and the severity of the footpad dermatitis (**FPD**) and the hock burns (**HB**). The FPD was scored as follows: 0 = no lesion, slight discoloration of the skin or healed lesion; 1 = mild lesion, superficial discoloration of the skin and hyperkeratosis; and 2 = severe lesion, affected epidermis, blood scabs, hemorrhages, and severe swelling of the skin (Ekstrand et al., 1997). Differently, the HB were scored as follows: 0 = no lesion; 1 = superficial, attached (single) lesion or several single superficial or deep lesions ≤0.5 cm; 2 = deep lesion >0.5 cm to ≤1 cm or superficial lesion >0.5 cm; 3 = deep lesion >1.0 cm; 4 = whole hock extensively altered (Louton et al., 2020).

### Organ Weights

On d 3 and 10, all the birds were individually weighed and 16 broilers/diet (2 birds/pen) were chosen to be euthanized. On d 3, the chicks of the pen with the highest LW were selected. On d 10, the birds that were closest to the average pen LW were, instead, chosen. The selected chicks were euthanized by cervical dislocation and bleeding. The weight of the liver and the pancreas were immediately recorded and the organ weights were expressed as a percentage of the LW.

### Histomorphological Investigations

**Figure 1:**
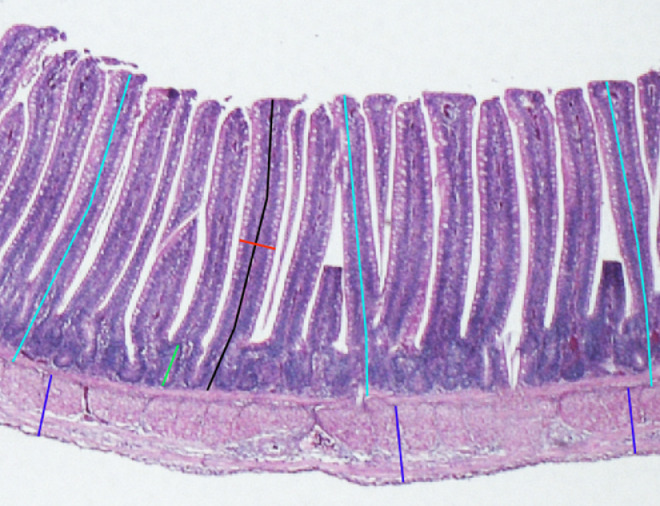
Morphometric measurements of villus height (black bar), crypt depth (green bar), villus width (red bar), mucosal thickness (light blue bars), and muscular thickness (dark blue bars) on a jejunum from a 10-day-old broiler chick fed T1 diet (hematoxylin & eosin, 2.5×).

After bird euthanasia (d 3 and 10), intestinal samples (approximately 2 cm in length) of the duodenum and jejunum were excised and flushed with 0.9% saline to remove all the content. The collected segments of the intestine were the loop of the duodenum and the tract before Meckel's diverticulum (jejunum). Liver and bursa of Fabricius were also sampled. The gut and organ samples were fixed in a 10% buffered formalin solution for morphometric and histopathological analyses, respectively. In particular, the fixed tissues were routinely embedded in paraffin wax blocks, sectioned at 5 μm thickness, mounted on glass slides, and stained with hematoxylin & eosin (**H&E**). One slide per each intestinal segment was examined by light microscopy and each slide was captured with a Nikon DS-Fi1 digital camera (Nikon Corporation, Minato, Tokyo, Japan) coupled to a Zeiss Axiophot microscope (Carl Zeiss, Oberkochen, Germany) using a 2.5 × objective lens. The NIS-Elements F software was used for image capturing and morphometric analysis was performed by Image-Pro Plus software (6.0 version, Media Cybernetics, MD, USA). The evaluated morphometric indices (Figure 1) were the villus height (**Vh**, from the tip of the villus to the crypt), the crypt depth (**Cd**, from the base of the villus to the submucosa), the villus width (**Vw**), and the villus height to crypt depth (Vh/Cd) ratio (Laudadio et al., 2012). The villus surface area (**VSA**) was calculated according to the following formula: (2π)(Vw/2)(Vh) (Sakamoto et al., 2000). These morphometric analyses were performed on 10 well-oriented and intact villi and 10 crypts chosen from the duodenum and jejunum (Qaisrani et al., 2014). The mucosal (**MT**) and muscular (**MuT**) thickness were also measured on 3 standardized points of the gut mucosal and muscular layers per each captured field (Figure 1).

In addition, the following histopathological alterations were evaluated: hepatocyte degeneration and lymphoid tissue activation in the liver, and follicular depletion in the bursa of Fabricius. Gut histopathological findings were separately assessed for mucosa (inflammatory infiltrates) and submucosa (inflammatory infiltrates and Gut-Associated Lymphoid Tissue [**GALT**] activation) for each segment. The observed histopathological alterations were evaluated using a semiquantitative scoring system as follows: absent (score = 0), mild (score = 1), moderate (score = 2), and severe (score = 3). The total score of each gut segment was obtained by adding up the mucosa and submucosa scores.

### Enzymatic Activity

After bird euthanasia, a segment of duodenum and jejunum was also collected, snap-frozen in liquid nitrogen, and stored at −20°C until analysis for the activities of the brush border enzymes (sucrase, maltase, and aminopeptidase), trypsin, and chymotrypsin. In particular, the duodenal and jejunal tissues were processed and the obtained homogenates were transferred into Eppendorf tubes (1.5 mL) in duplicates and stored at −20°C for subsequent enzyme analyses. The concentration of protein in both the duodenal and jejunal tissue homogenates was measured using the Comassie dye-binding procedure described by Bradford (1976). The aminopeptidase activities were assessed using methods described by Caviedes-Vidal and Karasov (2001) using spectrophotometric techniques, while trypsin and chymotrypsin activities were measured using N-benzoyl-L-arginine-ethylester (**BAME**) and N-benzoyl-L-tyrosine-ethylester (**BTEE**) as the respective substrates (Bergmeyer et al., 1974). Finally, the sucrase and maltase activities were assayed colorimetrically at 540 nm using a spectrophotometer by measuring μ moles of glucose released per min per g of tissue from sucrose and maltose, respectively (Dahlqvist, 1964; Palo et al., 1995; Uni et al., 1999).

### Gene Expression Analyses

In an aseptic environment, a segment of duodenum and jejunum was also cut off and put into a 2 mL grinding tube containing RNA Later (Sigma Aldrich, St Louis, MO). After 24 h at +4°C, RNA Later was removed and the samples were stored at −80°C until further analysis. Total RNA was extracted separately from the duodenum and jejunum of each animal using TRIzol reagent (Invitrogen, Carlsbad, CA) according to the manufacturer's instructions. The RNA quality of every sample was quantified by Nanodrop 1000 spectrophotometer (Thermo Fisher Scientific Inc., Wiington, DE) and the ratio (OD260:OD280) ranged from 1.7 to 2.1. Then, samples were analyzed separately for dietary treatments (C, T1, T2), intestinal segment (duodenum and jejunum), and experimental time (d 3 and 10). Within each group, RNA obtained from 4 chickens was pooled, to analyze 4 pooled samples per group. Afterward, 1.5 μg of total RNA for each pool was reverse transcribed to cDNA by using the iScript cDNA Synthesis Kit (Bio-Rad Laboratories, Inc., CA, USA) according to manufacturer protocol and the cDNA was stored at −20°C.

Quantitative real-time PCR was performed using a 7500 Real Time PCR system (Applied Biosystems, Waltham, MA) in a 20-μL reaction mixture containing 2 μL of cDNA, 10 μL of SYBR Green Supermix kit (Bio-Rad Laboratories, Inc., CA) and 0.1 μL of forward and reverse primers (40 mM) of the selected genes. Primers used for selected genes (IL-2, IL-4, TNF-α, INF-γ, MUC-2, ZO-1, and CL-1) were designed based on the available sequences in GenBank and synthetized by Macrogen Inc. (Amsterdam, the Netherlands) (Table 3). Thermal conditions for performing qPCR were as follows: initial incubation at 95°C for 30 s; 40 cycles of denaturation at 95°C for 15 s and annealing/extension at 60°C for 60 s followed by a melting curve analysis (65–95°C with 0.5°C increments at 2–5 s/step). The relative standard curve method was performed using ß-actin and glyceraldehyde-3-phosphate (**GADPH**) as internal control genes to normalize for RNA abundance. Each reaction was run in duplicate. Efficiency curves were performed for each primer set using log10 diluted cDNA to obtain efficiency-corrected relative quantification. Amplification efficiency between 90 and 110% was considered good with correlation coefficient *R*^2^ values of 0.99 (Rebrikov and Trofimov, 2006).

### Statistical Analysis

Statistical analysis was performed using both the IBM SPSS Statistics v. 26.0 (IBM, Armonk, NY) and the R software version 4.0.4 (R Foundation for Statistical Computing, Vienna, Austria; http://www.r-project.org). The Shapiro-Wilk test was used to check dependent variables or residuals for normality. Growth performance and organ weights data were analyzed by 1-way ANOVA using the following model:

$$Y_{\text{ij}}=\mu +D_{i}+\varepsilon_{\text{ij}}$$

where Y_ij_ is the observation; μ is the overall mean; D_i_ is the effect of diet (C, T1, T2); and ε_ij_ is the residual error. The assumption of equal variances was assessed by Levene's homogeneity of variance test. If such an assumption did not hold, the Brown-Forsythe statistic was performed to test for the equality of group means instead of the F one. Pairwise multiple comparisons were performed to test the difference between each pair of means (Tukey's test and Tamhane's T2 in the cases of equal variances assumed or not assumed, respectively). The PALS, FPD, and HB scores were analyzed using the Kruskal-Wallis test (post hoc test: Dunn's Multiple Comparison test). Histological scores were also analyzed by 1-way ANOVA test or the corresponding nonparametric Kruskal-Wallis test and Tukey post hoc tests. Gut histomorphological findings were analyzed by fitting a general linear model (**GLM**, SPSS). In particular, the GLM allowed the morphometric indices (Vh, Cd, Vh/Cd, Vw, VSA, MT, and MuT) to depend on 3 fixed factors (diet, intestinal segment, and the interaction between the diet and intestinal segment) per each age separately (3 and 10 d). Similarly, gut enzymatic activities were analyzed by fitting a generalized linear mixed model (**GLMM**, SPSS). In particular, the GLMM allowed the enzymatic activities (sucrase, maltase, aminopeptidase, trypsin, and chymotrypsin) to depend on 3 fixed factors (diet, intestinal segment, and the interaction between the diet and the intestinal segment) per each age separately (d 3 and 10). In both the statistical models, the bird was included as a random effect to account for repeated measurements, and the interactions between the levels of the fixed factors were evaluated using pairwise contrasts.

As far as the intestinal gene expression data are concerned, Microsoft Excel was used to convert the quantification cycle values to linear units called relative normalized expression according to Ahmed and Kim (2018) and Colombino et al. (2021). Samples with relative normalized expression >10 were identified as potential outliers and excluded from the analysis. The Shapiro-Wilk test was then used to test the normality of the data distribution and a robust ANOVA test was performed by the trimmed means method. The ANOVA test allowed the evaluated variables to depend on 3 fixed factors (diet, intestinal segment, and the interaction between diet and intestinal segment). The interactions were evaluated by pairwise comparisons (R software version 4.0.4).

The results were expressed as the mean (growth and organ weights data, intestinal gene expression findings, and PALS, FPD, and HB scores) or least square mean (gut histomorphological findings and enzymatic activities) and standard error of the mean (**SEM**). *P* values ≤0.05 were considered statistically significant. A statistical trend was considered for 0.05 < *P* ≤ 0.10.

## RESULTS

### Growth Performance

The growth performance of the broiler chickens are summarized in Table 5. The birds readily accepted the experimental diets and remained healthy throughout the whole growth trial. Furthermore, the mortality was similar among the 3 dietary treatments (*P* > 0.05, Table 4). The LW on d 3 and 7 showed a positive statistical trend, with the T1 broilers tending to have higher LW than the other groups (*P* < 0.10, Table 5). On the contrary, the LW at 10, 14, 21, 28, 35, and 42 d of age was not affected by dietary treatments (*P* > 0.05, Table 4). Similarly, the T1 broilers tended to show a higher ADG than the other groups in the prestarter and starter phases (*P* ≤ 0.10) but not in the other growth phases or in the whole experimental period (*P* > 0.10) (Table 4). Moreover, the dietary treatments did not influence DFI and the FCR in all the growth phases (*P* > 0.05, Table 5).

**Table 4:** Oligonucleotide primers used for quantitative reverse transcription-PCR.^1^

Type	RNA target	Primer sequence	GenBank accession no.
Reference gene	ß-Actin	F:5′-GAGAAATTGTGCGTGACATCA-3′ R:5′-CCTGAACCTCTCATTGCCA-3′	L08165.1
	GADPH	F:5′-GGTGGTGCTAAGCGTGTTAT-3′ R:5′-ACCTCTGTCATCTCTCCACA-3′	K01458
Target genes	TNF-α	F:5′-CCCATCTGCACCACCTTCAT-3′ R:5′-CATCTGAACTGGGCGGTCAT-5′	AY765397.1
	INF-γ	F:5′-AGCTGACGGTGGACCTATTATT-3′ R:5′-GGCTTTGCGCTGGATTC-3′	Y07922.1
	IL-2	F:5′-TCTGGGACCACTGTATGCTCT-3′ R:5′-ACACCAGTGGGAAACAGTATCA-3′	AF000631
	IL-4	F:5′-CTTCCTCAACATGCGTCAGC-3′ R:5′-TGAAGTAGTGTTGCCTGCTGC-3′	AJ621735
	MUC-2	F: 5′-ACTCCTCCTTTGTATGCGTGA-3′ R: 5′-GTTAACGCTGCATTCAACCTT-3′	NM.001318434.1
	CL-1	F:5′-GTGTTCAGAGGCATCAGGTATC-3′ R:5′-GTCAGGTCAAACAGAGGTACAA-3′	NM_001013611.2
	ZO-1	F:5′-GCCAACTGATGCTGAACCAA-3′ R:5′-GGGAGAGACAGGACAGGACT-3′	XM_015278975

1 Abbreviations: CL, claudin; F, forward primer; GAPDH, glyceraldehyde-3-phosphate; IL, interleukin; INF, interferon; MUC, mucin; R, reverse primer; TNF, tumor necrosis factor; ZO, zonula occludens.

**Table 5:** Effects of the coprocessed yeast and soybean meal on growth performance of broiler chickens (*n* = 8/dietary treatment).

Items^1^	Age	Diets^2^			SEM	*P* value
		C	T1	T2		
Mortality, %	0–42 d	1.04	0.52	1.56	0.45	0.451
LW, g	0 d	39.22	39.23	39.32	0.05	0.686
	3 d	65.60^B^	68.69^A^	66.28^B^	0.61	0.089
	7 d	121.82^B^	129.47^A^	123.84^B^	1.50	0.094
	10 d	177.10	185.96	180.59	1.78	0.121
	14 d	283.81	293.93	289.64	2.88	0.370
	21 d	552.57	567.91	564.27	5.46	0.508
	28 d	930.97	956.53	945.32	10.37	0.622
	35 d	1433.35	1461.18	1451.50	17.54	0.819
	42 d	1988.34	2025.64	2032.89	23.04	0.717
ADG, g/d	0–3 d	8.44^B^	9.39^A^	8.44^B^	0.21	0.100
	4–10 d	15.59^B^	16.71^A^	15.84^B^	0.21	0.075
	11–21 d	33.97	34.63	34.38	0.46	0.852
	22–35 d	62.49	63.85	62.17	0.95	0.760
	36–42 d	80.27	79.73	82.02	1.54	0.896
	0–42 d	46.41	47.30	47.47	0.55	0.718
DFI, g/d	0–3 d	6.79	7.99	7.11	0.24	0.111
	4–10 d	20.75	22.22	21.17	0.31	0.131
	11–21 d	59.31	60.62	58.95	1.17	0.842
	22–35 d	99.92	101.65	105.26	1.66	0.424
	36–42 d	143.10	139.92	144.91	1.54	0.429
	0–42 d	77.11	77.87	79.16	0.88	0.680
FCR, g/g	0–3 d	0.79	0.84	0.84	0.01	0.173
	4–10 d	1.32	1.33	1.31	0.01	0.658
	11–21 d	1.76	1.75	1.71	0.03	0.841
	22–35 d	1.61	1.59	1.70	0.03	0.266
	36–42 d	1.81	1.77	1.78	0.04	0.891
	0–42 d	1.66	1.64	1.68	0.02	0.719

1 Abbreviations: ADG, average daily gain; DFI, daily feed intake; FRC, feed conversion ratio; LW, live weight. Means with different superscript letters (A, B) indicate statistical tendencies among the experimental treatments (*P* ≤ 0.10).

2 C = control diet; T1 = 20% inclusion of the coprocessed yeast and soybean meal in the prestarter phase, 10% in the starter phase and 5% in the grower phase; T2 = 5% inclusion of the coprocessed yeast and soybean meal in the prestarter, starter, and grower phases; SEM = standard error of the mean.

As far as the LW variations within the groups are considered (Table 6), both the CV and the uniformity of the pYSM groups was numerically improved when compared to the C-fed birds, displaying 7% less CV and 16% more uniformity at the end of the experimental trial.

**Table 6:** Effects of the coprocessed yeast and soybean meal on uniformity and coefficient of variation of live weight of broiler chickens.

Age	Parameters	C-fed birds	pYSM-fed birds^1^
D 0	Uniformity (%)	82.0	71.0
	CV (%)	7.48	9.56
D 14	Uniformity (%)	49.0	56.0
	CV (%)	13.46	12.17
D 28	Uniformity (%)	52.0	53.0
	CV (%)	14.75	13.87
D 42	Uniformity (%)	46.0	55.0
	CV (%)	15.56	14.50

1 The results are expressed as mean of uniformity and CV of T1 and T2 groups. C = control diet; T1 = 20% inclusion of the coprocessed yeast and soybean meal in the prestarter phase, 10% in the starter phase, and 5% in the grower phase; T2 = 5% inclusion of the coprocessed yeast and soybean meal in the prestarter, starter and grower phases; CV = coefficient of variation; pYSM = coprocessed yeast and soybean meal. The sex ratio in all the dietary treatments was analogous and very close to 1:1 (50% males and 50% females).

### Litter Quality Evaluation and Feet and Hock Health Assessment

The litter used to rear the broiler chickens fed the C diet was characterized by a statistical tendency of higher average PALS scores compared to the T1 and T2 groups (C: 22.25 ± 1.82; T1: 17.25 ± 1.40; T2: 18.13 ± 1.13; *P* = 0.079).

Regarding the feet and hock health assessment, at d 35, the FPD and HB scores were unaffected by the pYSM inclusion (*P* > 0.05). In particular, the majority of the birds showed FPD scores ranging from 0 (C: 100%; T1: 98.73%; T2: 99.36%) to 1 (C: 0%; T1: 1.27%; T2: 0.64%). Differently, the HB scores resulted to be 0 (C: 100%; T1: 98.73%; T2: 98.73%), 1 (C: 0%; T1: 0%; T2: 0.64%) and 2 (C: 0%; T1: 0%; T2: 0.64%). At d 42, the FPD and HB scores were unaffected by dietary treatments (*P* > 0.05). In particular, the majority of the birds showed FPD scores ranging from 0 (C: 100%; T1: 98.73%; T2: 99.36%) to 1 (C: 0%; T1: 1.27%; T2: 0.64%). Differently, the HB scores resulted to be 0 (C: 89.24%; T1: 93.04%; T2: 89.81%), 1 (C: 9.49%; T1: 6.96%; T2: 8.92%), 2 (C: 1.27%; T1: 0%; T2: 0.64%) and 3 (C: 0%; T1: 0%; T2: 0.64%).

### Organ Weights

The organ weights of the broiler chickens are summarized in Table 7. On d 3, the birds fed the T1 diet showed higher LW (by 5.34%; *P* = 0.02) when compared to the C group (Table 7). However, the relative pancreas and liver weights (expressed as percentages of LW) were similar among the 3 dietary treatments at d 3 (*P* > 0.10, Table 7). On d 10, the broiler chickens fed the T1 diet also showed higher LW (by 4.97%) in comparison with the C group (*P* < 0.05, Table 7). Differently, the relative pancreas and liver weights were similar among the dietary treatments (*P* > 0.05, Table 7).

**Table 7:** Effects of the coprocessed yeast and soybean meal on organ weights of broiler chickens (*n* = 16/dietary treatment).

Items^1^	Age	Diets^2^			SEM	*P* value
		C	T1	T2		
LW, g	3 d	74.94^a^	78.94^b^	76.50^ab^	0.60	0.020
	10 d	175.06^a^	183.76^b^	177.01^ab^	1.45	0.032
Pancreas weight, %LW	3 d	0.44	0.48	0.47	0.01	0.214
	10 d	0.48	0.46	0.45	0.01	0.573
Liver weight, %LW	3 d	4.87	5.21	5.13	0.10	0.343
	10 d	3.61	3.84	3.83	0.06	0.229

1 Abbreviations: LW, live weight; SEM, standard error of the mean.

2 C = control diet; T1 = 20% inclusion level of the coprocessed yeast and soybean meal in the prestarter phase, 10% in the starter phase and 5% in the grower phase; T2 = 5% inclusion level of the coprocessed yeast and soybean meal in the prestarter, starter, and grower phases. Means with different superscript letters indicate significant differences (a, b; *P* ≤ 0.05) among the experimental treatments.

### Histomorphological Investigations

The gut morphology of the broiler chickens is summarized in Table 8. Dietary pYSM inclusion did not influence the intestinal morphometric indices in both the 3-day and 10-day-old broiler chicks (*P* > 0.05). However, all the morphometric indices were greater in the duodenum when compared to the jejunum (*P* < 0.05).

**Table 8:** Effects of the coprocessed yeast and soybean meal on gut morphology of the broiler chickens at d 3 and 10 (*n* = 16/dietary treatment).

	Diet (D)^2^			Intestinal segment (IS)^3^		SEM		*P* value		
Items^1^	C	T1	T2	DU	JE	D	IS	D	IS	DxIS
D 3										
Vh (mm), mean	0.57	0.61	0.57	0.73	0.46	0.02	0.02	0.400	0.001	0.996
Cd (mm), mean	0.07	0.06	0.07	0.07	0.06	0.01	0.01	0.801	0.003	0.920
Vh/Cd, mean	10.19	9.40	8.59	11.43	7.33	0.90	0.75	0.099	0.001	0.658
Vw (mm), mean	0.07	0.07	0.07	0.07	0.07	0.01	0.01	0.323	0.042	0.383
VSA (mm^2^), mean	0.13	0.13	0.12	0.17	0.09	0.01	0.01	0.303	0.001	0.731
MT (mm), mean	0.70	0.73	0.70	0.87	0.56	0.02	0.02	0.478	0.001	0.925
MuT (mm), mean	0.11	0.12	0.10	0.12	0.01	0.01	0.01	0.553	0.001	0.411
D 10										
Vh (mm), mean	0.82	0.80	0.76	1.07	0.49	0.03	0.02	0.055	0.001	0.606
Cd (mm), mean	0.06	0.09	0.09	0.10	0.06	0.02	0.02	0.574	0.001	0.783
Vh/Cd, mean	12.90	12.48	12.05	16.39	8.63	0.02	0.02	0.351	0.001	0.955
Vw (mm), mean	0.10	0.09	0.10	0.10	0.09	0.01	0.01	0.635	0.001	0.705
VSA (mm^2^), mean	0.27	0.24	0.24	0.37	0.14	0.01	0.01	0.145	0.001	0.455
MT (mm), mean	0.93	0.89	0.82	1.21	0.55	0.03	0.02	0.225	0.001	0.725
MuT (mm), mean	0.15	0.15	0.13	0.17	0.12	0.01	0.01	0.320	0.001	0.800

1 Abbreviations: Cd, crypt depth; MT, mucosa thickness; MuT, muscular thickness; SEM, standard error of the mean; Vh, villus height; VSA, villus surface area; Vw, villus width.

2 C = control diet; T1 = 20% inclusion level of the coprocessed yeast and soybean meal in the prestarter phase, 10% in the starter phase and 5% in the grower phase; T2 = 5% inclusion level of the coprocessed yeast and soybean meal in the prestarter, starter, and grower phases.

3 DU = duodenum; JE = jejunum.

**Figure 2:**
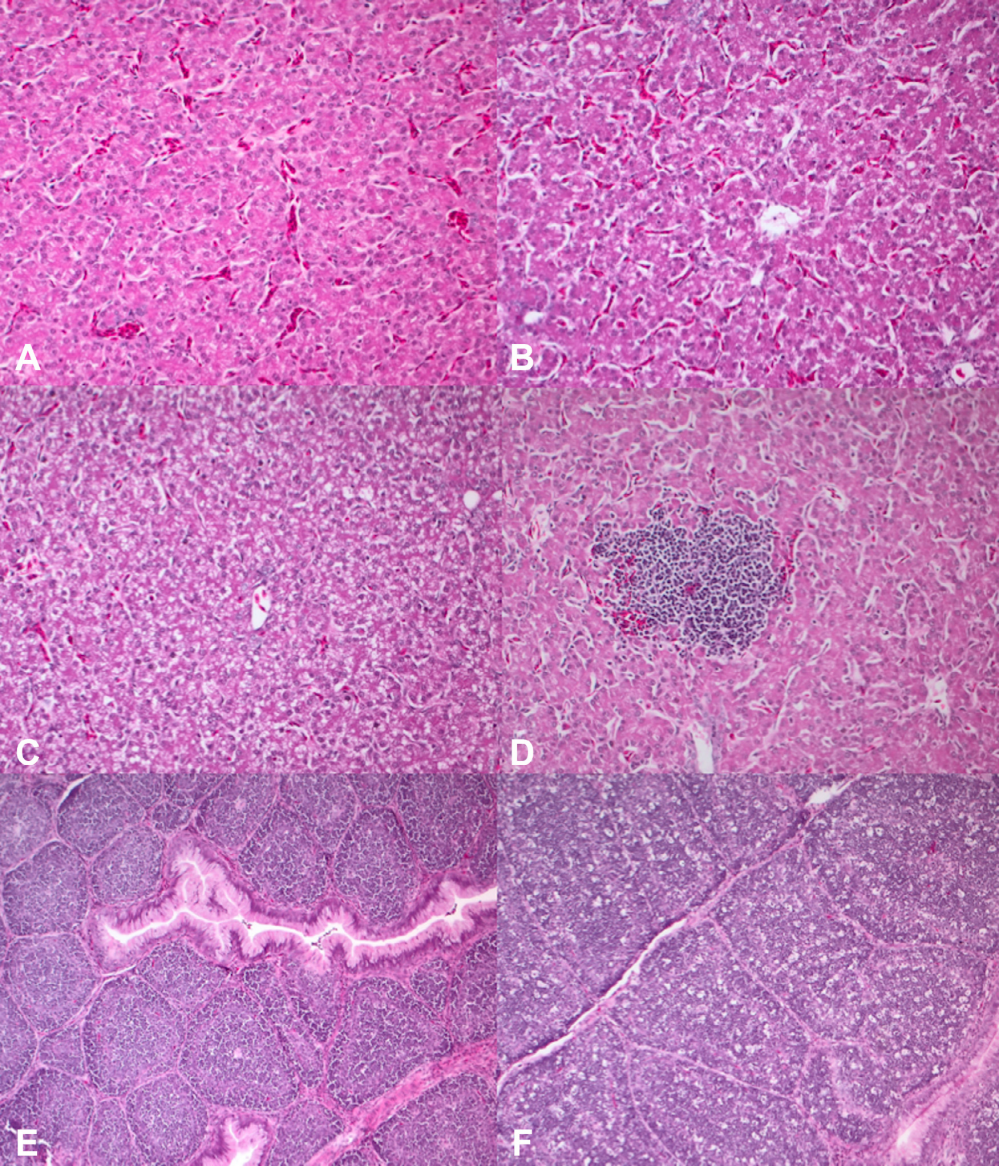
Main histopathological findings in chicks's liver and bursa of Fabricius. (A) A normal liver (grade 0), 20×, hematoxylin & eosin (H&E). (B) Mild and multifocal vacuolar degeneration (grade 1), 20×, H&E. (C) Moderate and multifocal vacuolar degeneration (grade 2), 20×, H&E. (D) Mild and multifocal lymphoplasmacytic inflammation (grade 1), 20×, H&E. (E) A normal bursa of Fabricius (grade 0), 10×, hematoxylin & eosin (H&E). (F) Bursa of Fabricius, mild and multifocal follicular depletion, 10×, H&E.

**Figure 3:**
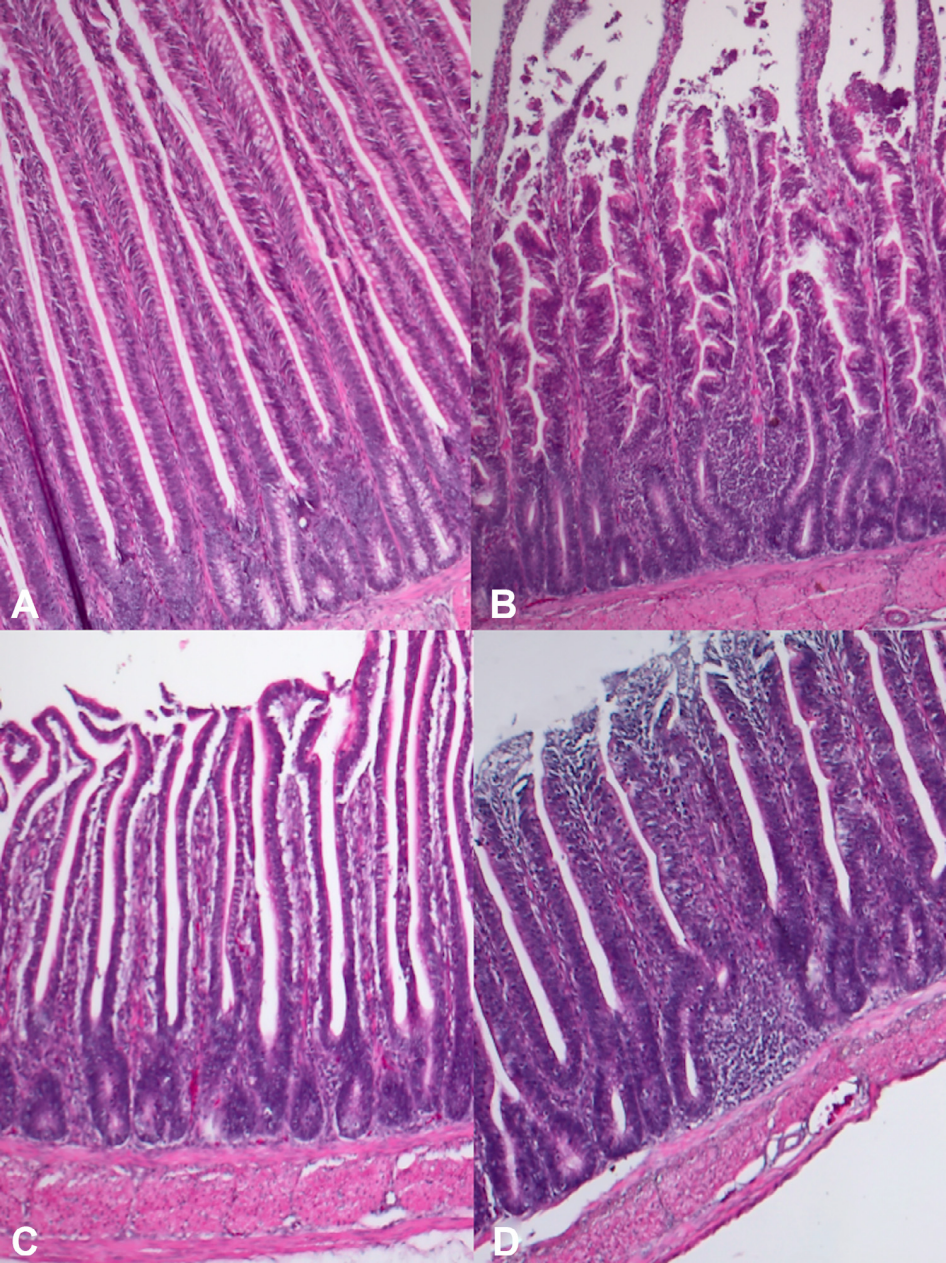
Main histological findings of chick's gut. (A) A normal duodenum (grade 0), 10×, hematoxylin & eosin (H&E). (B) Mild and multifocal lymphoplasmacytic duodenitis (grade 1), 10×, H&E. (C) A normal jejunum (grade 0), 10, H&E. (D) Mild and multifocal lymphoplasmacytic jejunitis (grade 1), 10×, H&E.

Regarding the evaluation of the main organs, no significant differences were recorded among the dietary treatments both on d 3 and 10 (*P* > 0.05) (Table 9). Regardless of diet, the liver showed mild and multifocal vacuolar degeneration (Figure 2A–C) as well as mild and multifocal lymphoplasmacytic infiltrates (Figure 2D). Bursa of Fabricius presented from absent to mild, multifocal follicular depletion (Figures 2E and F). Gut showed from absent to mild, multifocal lymphoplasmacytic infiltrates (Figure 3).

**Table 9:** Effects of the coprocessed yeast and soybean meal on histopathological scores of liver, gut, and bursa of Fabricius of the broiler chickens at d 3 and 10 (*n* = 16/dietary treatment).

	Diet^1^			SEM	*P* value
Items	C	T1	T2		
D 3					
Liver inflammation, mean	0.600	0.667	0.562	0.071	0.844
Liver degeneration, mean	1.300	1.233	1.719	0.085	0.064
Bursa depletion, mean	0.656	0.667	0.767	0.077	0.877
Duodenum inflammation, mean	0.094	0.094	0.094	0.038	>0.999
Jejunum inflammation, mean	0.125	0.156	0.312	0.039	0.269
D 10					
Liver inflammation, mean	0.625	0.812	0.812	0.059	0.433
Liver degeneration, mean	0.156	0.469	0.406	0.077	0.180
Bursa depletion, mean	0.875	1.233	1.250	0.076	0.130
Duodenum inflammation, mean	0.250	0.281	0.469	0.070	0.445
Jejunum inflammation, mean	0.406	0.656	0.469	0.085	0.482

1 Abbreviations: C, control diet; T1, 20% inclusion level of the coprocessed yeast and soybean meal in the prestarter phase, 10% in the starter phase and 5% in the grower phase; T2, 5% inclusion level of the coprocessed yeast and soybean meal in the prestarter, starter, and grower phases.

### Gene Expression Analysis

**Figure 4:**
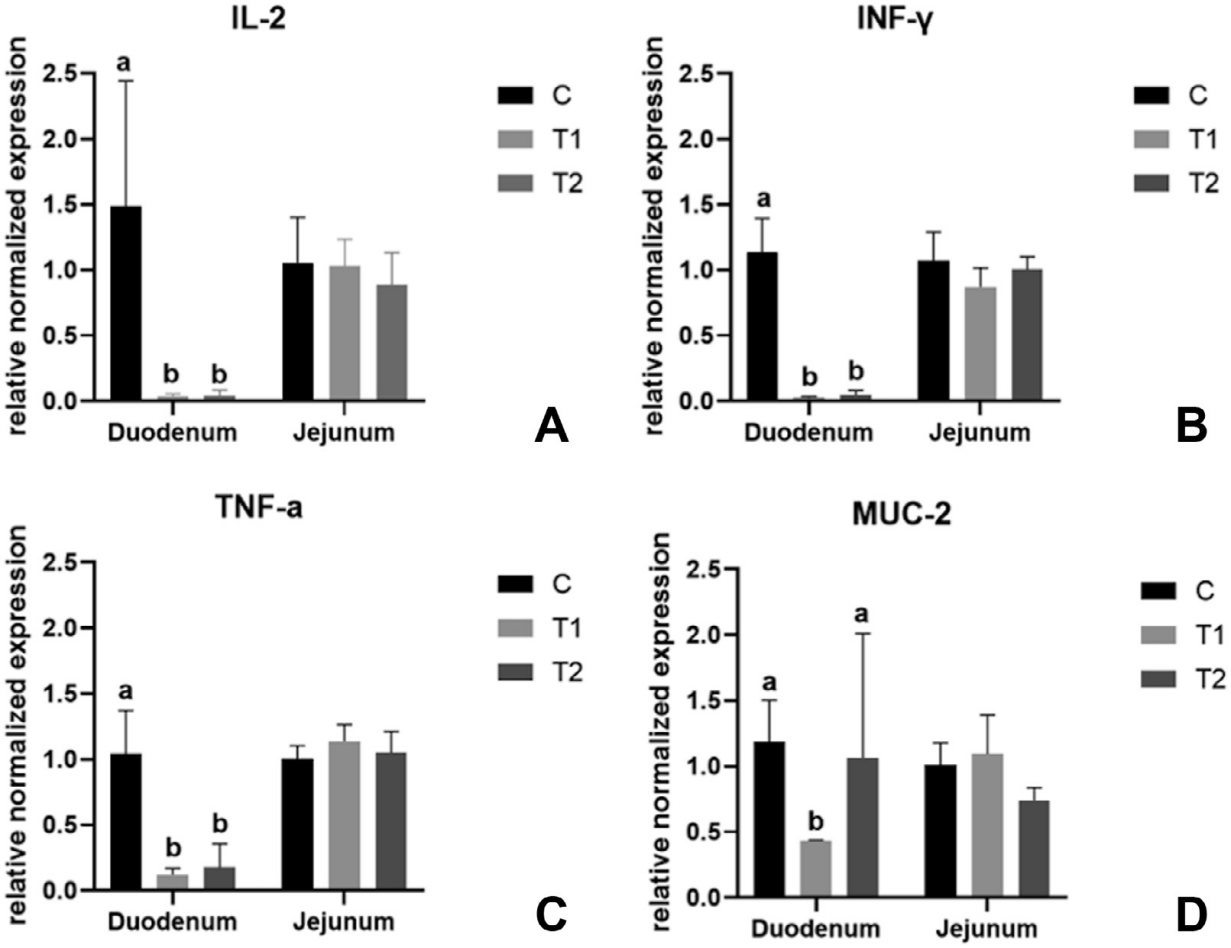
Effects of the coprocessed yeast and soybean meal on the relative mRNA expression of cytokines and mucin-related genes in duodenum and jejunum of 3-day-old broiler chickens depending on the interaction between the diet and the gut segment (*n* = 4/dietary treatment). Graph bars with different superscript letters (a, b, c) denote significant differences among the experimental treatments. C = control diet; T1 = 20% inclusion of the coprocessed yeast and soybean meal in the prestarter phase, 10% in the starter phase and 5% in the grower phase; T2 = 5% inclusion of the coprocessed yeast and soybean meal in the prestarter, starter, and grower phases.

The cytokine transcription levels in 3-day-old broiler chickens are summarized in Table 10 and Figure 4. The expression of IL-2 was influenced by dietary treatments (*P* = 0.054) and intestinal segment (*P* = 0.038), being 63.8% lower in the T1 and T2 birds when compared to the C group, and being 1.9 times higher in the jejunum than in the duodenum (Table 10). Furthermore, INF-γ and TNF-α transcription levels were influenced by the interaction between diet and intestinal segment (*P* = 0.058 and *P* < 0.001, respectively) while IL-2 showed a statistical tendency (*P* = 0.083) (Figure 4). In particular, both T1 and T2 groups had lower proinflammatory cytokines when compared to C group in the duodenum (*P* ≤ 0.05), but not in the jejunum (*P* > 0.05) (Table 10). On the contrary, IL-4 was not influenced by diet (*P* > 0.05), but it only depends on the gut segment (*P* = 0.010), being higher in the jejunum than in the duodenum (Table 10). Furthermore, MUC-2 was influenced by the interaction between diet and intestinal segment (*P* = 0.016), being 30.9 and 18.18% lower in the duodenum of T1 birds compared to C and T2 groups, respectively. Moreover, ZO-1 showed a statistical tendency among dietary treatments at d 3, being lower in T1 compared to C and T2 groups (*P* = 0.085). Differently, no significant differences among the dietary treatments and between the intestinal segments were identified for all the evaluated cytokines, mucin, and tight junction transcription levels at d 10 (*P* > 0.05, Table 10).

**Table 10:** Effects of the coprocessed yeast and soybean meal on relative mRNA expression of cytokines, mucin, and tight junction-related genes in small intestine of the broiler chickens at d 3 and 10 (*n* = 4/dietary treatment).

	Diet (D)^2^			Intestinal segment (IS)^3^		SEM^4^		*P* value		
Items^1^	C	T1	T2	DU	JE	D	IS	D	IS	D × IS
D 3										
IL-2	1.27^a^	0.46^b^	0.46^b^	0.52	0.98	0.16	0.12	0.054	0.038	0.083
IL-4	1.01	0.51	0.57	0.27	1.11	0.13	0.11	0.423	0.010	0.240
INF-γ	1.10^a^	0.45^b^	0.52^b^	0.40	0.98	0.11	0.09	0.020	0.001	0.058
TNF-α	1.02^a^	0.63^b^	0.61^b^	0.45	1.06	0.06	0.05	0.005	0.001	0.001
MUC-2	1.10	0.76	0.90	0.89	0.95	0.15	0.12	0.069	0.774	0.016
ZO-1	1.00^a^	0.79^b^	0.91^a^	0.94	0.86	0.14	0.12	0.085	0.682	0.105
CL-1	1.20	1.10	1.62	1.11	1.51	0.38	0.31	0.706	0.387	0.119
D 10										
IL-2	1.46	2.37	1.86	2.09	1.71	0.39	0.32	0.322	0.418	0.793
IL-4	1.34	1.57	1.67	1.64	1.41	0.21	0.17	0.382	0.362	0.593
INF-γ	1.40	1.50	1.68	1.49	1.56	0.25	0.20	0.763	0.803	0.470
TNF-α	1.34	1.50	1.38	1.50	1.32	0.07	0.06	0.249	0.056	0.120
MUC-2	1.39	1.41	1.38	1.49	1.29	0.11	0.09	0.970	0.151	0.169
ZO-1	1.38	1.22	1.12	1.30	1.18	0.06	0.05	0.097	0.152	0.448
CL-1	1.49	1.64	1.79	1.65	1.63	0.23	0.19	0.676	0.961	0.857

1 Changes in each gene expression were normalized to ß-actin and GADPH. Abbreviations: CL, claudin; IFN, interferon; IL, interleukin; MUC, mucin 2; TNF, tumor necrosis factor; ZO, zonula occludens.

2 C: control diet; T1 = 20% inclusion of the coprocessed yeast and soybean meal in the prestarter phase, 10% in the starter phase and 5% in the grower phase; T2 = 5% inclusion of the coprocessed yeast and soybean meal in the prestarter, starter, and grower phases.

3 DU = duodenum; JE = jejunum.

4 SEM = standard error of the mean. Means with different superscript letters (a, b) within a row were found to be different at a significance level of *P* ≤ 0.05.

### Enzymatic Activity

**Figure 5:**
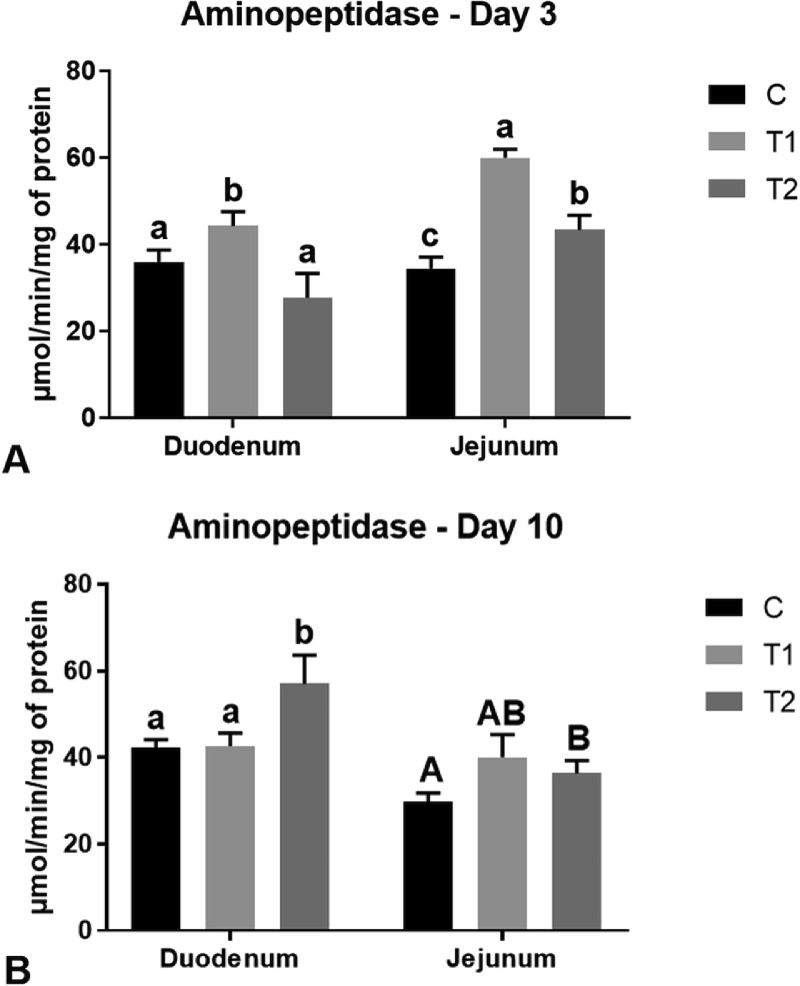
Effects of the coprocessed yeast and soybean meal inclusion on the aminopeptidase activity in duodenum and jejunum of (A) 3-day and (B) 10-day-old broiler chickens depending on the interaction between the diet and the gut segment. Graph bars with different superscript letters denote significant differences (a, b, c; *P* ≤ 0.05) or statistical tendencies (A, B; *P* ≤ 0.10) among the experimental treatments. C = control diet; T1 = 20% inclusion of the coprocessed yeast and soybean meal in the prestarter phase, 10% in the starter phase and 5% in the grower phase; T2 = 5% inclusion of the coprocessed yeast and soybean meal in the prestarter, starter, and grower phases.

Data regarding the intestinal enzymatic activities of the broiler chickens are reported in Table 11 and Figure 5. At both 3 and 10 d of age, the activities of both sucrase and maltase were not influenced by dietary pYSM inclusion (*P* > 0.05), but they only depend on the intestinal segment (*P* < 0.001). In particular, higher enzymatic activities were observed in the jejunum when compared to the duodenum (*P* < 0.001). However, there was a significant interaction between the diet and the gut segment on aminopeptidase activity (*P* < 0.001). Indeed, the T1-fed broiler chickens showed greater aminopeptidase activity than the other groups in both the duodenum and the jejunum (*P* < 0.05), while T2-fed broiler chicken had a higher aminopeptidase activity than C-fed ones only in the jejunum (*P* = 0.036), but not in the duodenum (*P* < 0.05, Figure 5). At 10 d of age, there was a tendency of interaction effect between dietary treatment and the intestinal segment (*P* = 0.051). Indeed, the T2-fed broiler chickens had a greater aminopeptidase activity than the other groups in the duodenum (*P* < 0.05), while it tended to be higher than C group (*P* = 0.063), but not than T1 group (*P* > 0.10) in the jejunum (Figure 5). Finally, the activities of either the trypsin or the chymotrypsin were not influenced by any of the considered variables (*P* > 0.05, Table 11).

**Table 11:** Effects of the coprocessed yeast and soybean meal on the enzymatic activities in duodenum and jejunum of the broiler chickens at d 3 and 10 (*n* = 16/dietary treatment).

	Diet (D)^1^			Intestinal segment (IS)^2^		SEM^3^		*P* value		
Items	C	T1	T2	DU	JE	D	IS	D	IS	D × IS
D 3										
Sucrase, µmol/min/g tissue	7.54	7.63	7.60	5.17^a^	10.01^b^	0.09	0.07	0.706	<0.001	0.520
Maltase, µmol/min/g tissue	140.67	139.02	137.40	91.69^a^	186.37^b^	2.68	2.19	0.519	<0.001	0.464
Aminopeptidase, µmol/min/mg protein	35.27^a^	52.17^b^	35.60^a^	36.04^a^	45.99^b^	2.86	1.95	<0.001	<0.001	<0.001
Trypsin, µmol/min/mg protein	0.12	0.14	0.16	0.17	0.12	0.03	0.03	0.664	0.079	0.102
Chymotrypsin, µmol/min/mg protein	0.53	0.50	0.51	0.48	0.55	0.06	0.06	0.942	0.457	0.872
D 10										
Sucrase, µmol/min/g tissue	16.80	16.20	16.19	10.01^a^	22.80^b^	0.52	0.60	0.640	<0.001	0.767
Maltase, µmol/min/g tissue	377.55	377.43	378.23	241.17^a^	514.30^b^	4.11	3.36	0.608	<0.001	0.836
Aminopeptidase, µmol/min/mg protein	35.96^a^	41.36^a^	46.72^b^	47.32^a^	35.37^b^	3.09	2.31	0.037	<0.001	0.051
Trypsin, µmol/min/mg protein	0.16	0.08	0.11	0.12	0.10	0.03	0.02	0.127	0.237	0.256
Chymotrypsin, µmol/min/mg protein	0.39	0.38	0.34	0.37	0.37	0.04	0.03	0.724	0.777	0.617

1 C: control diet; T1 = 20% inclusion of the co-processed yeast and soybean meal in the pre-starter phase, 10% in the starter phase and 5% in the grower phase; T2: 5% inclusion of the co-processed yeast and soybean meal in the pre-starter, starter and grower phases;

2 DU = duodenum; JE = jejunum;

3 SEM = standard error of the mean. Means with different superscript letters (a, b) within a row were found to be different at a significance level of *P* ≤ 0.05.

## DISCUSSION

To the author's knowledge, this is the first study evaluating the effects of a pYSM (produced by enzyme-treatment and thermomechanical coprocess of SBM and hydrolyzed yeast) on growth performance, organ weight, litter quality, feet and hock health, and gut development of broiler chickens. However, it is well known that highly processed soy protein increases both the availability and digestibility of nutrients, and yeast β-glucans and MOS can act as indirect growth promoters by increasing immunocompetence, thus having beneficial effects on growing chicks (Korver, 2012).

Dietary pYSM inclusion did not significantly influence the growth performance of the broiler chickens in the present study. Despite processed SBM utilization in starter diet having previously been reported to commonly improve broiler growth performance (Heger et al., 2016; Jahanian and Rasouli, 2016; Kim et al., 2016; Masey O'Neill et al., 2018; Chachaj et al., 2019), no effects can also be highlighted (Graham et al., 2002; Rasmussen et al., 2021). However, the T1 birds (with high dietary inclusion of pYSM) had the tendency to show higher LW when compared to the C group at d 3 (end of the prestarter feeding phase) and d 7 of age (middle of the starter feeding phase). Similarly, a tendency to show higher ADG in the prestarter and starter feeding phases was also identified in the broiler chickens fed the T1 diet in comparison with the other groups. The absence of a statistical significance may be probably related to the partial, inhomogeneous growth of the animals, as interestingly suggested by the findings observed after bird euthanasia. Indeed, the 3-day-old birds with the highest LW from the T1 diet displayed higher LW than the C group. Similarly, higher LW was also identified in the euthanized T1 10-day-old broilers with the LW closest to the average pen LW in comparison with the C animals. Such partial, inhomogeneous growth may potentially be attributed to the unexpected hot weather observed during the first 2 wk of the trial. Nevertheless, another fundamental aspect to highlight is that the broiler chickens of the current research were manipulated every 3 d during the first 2 wk of life, which may cause a potential, significant stress for them and, in turn, a reduction in the expected growth. In support of this hypothesis, Freeman and Manning (1979) observed that chicks subjected to frequent handling in the first 3 wk of age displayed a significant decrease in the growth rate. However, from a descriptive point of view, it is still interesting to highlight that the birds fed the T1 diet overall showed numerically higher LW, ADG, and DFI in the first 3 wk of life when compared to the other dietary treatments, and consequently displayed a slightly numerically lower overall FCR, thus representing potential, relevant aspects for the poultry industry. Furthermore, the identification of reduced LW variations (in terms of numerically lower CV and numerically higher uniformity) in the pYSM-fed groups needs also to be taken into account, as LW variations are still commonly observed in mixed-sex flocks, thus leading to decreased profitability due to devaluation of carcasses not complying with the processing plants and market specifications (Lundberg et al., 2021).

Moreover, dietary pYSM inclusion did not significantly affect the litter quality as well, as a confirmation of the similar leg health status observed among the experimental treatments. However, the litter used to rear the C birds had the tendency to show higher PALS scores when compared to the T1 and T2 diets, thus potentially suggesting the production of more aqueous feces in the C birds. Accordingly, dietary pYSM inclusion did not significantly influence neither the development nor the severity of the FPD and the HB in the broiler chickens during the last 2 wk of the experimental trial. Furthermore, the majority of the birds (around 90–99% per each dietary treatment) did not develop any feet or hock lesions, thus being in overall agreement with the average low PALS scores herein recorded (around 20%).

After euthanasia, the 3-day-old and 10-day-old broiler chickens fed the T1 diet showed higher LW when compared to the C group, but the relative weights of their pancreas and liver were not influenced by the utilization of the pYSM. The proportional increase in the relative pancreas weight has been reported to be an indicator of the physiological adaptive mechanism (hypertrophy and hyperplasia of pancreatic cells) of the organ to face the negative impact of the presence of antinutritional factors (i.e., trypsin inhibitors) in birds fed diets containing raw full-fat SBM (Rada et al., 2017; Lundberg et al., 2021). Indeed, the pancreas enlarges its surface area to produce more endogenous enzymes (especially the proteases) (Rada et al., 2017; Erdaw et al., 2018). As the pYSM herein tested is characterized by lower ANFs when compared to the conventional SBM, the unaffected relative pancreas weight appears to be reasonable. As far as the liver relative weight is concerned, its increase has been suggested as an indicator of better immune system in chickens (Zhou et al., 2009). Therefore, the absence of pYSM-related effects may suggest a similar systemic immune response in birds.

Independently of the age, feeding pYSM did not significantly influence the gut morphology of the broiler chickens, thus suggesting no pYSM-related negative effects on intestinal development, health, and functionality, and reasonably explaining the unaffected small intestine weights. These results are in contrast with the available literature, as previous studies reported that β-glucans contained in yeast can improve gut morphology (Teng et al., 2021). In particular, Morales-López et al. (2009), Ding et al. (2019), and Teng et al. (2021) found greater Vh and Cd in duodenum and jejunum/ileum after the administration of yeast β-glucans. Moreover, Cox et al. (2010) reported ameliorated Vh and Cd after β-glucans administration in *Eimeria*- or coccidian-challenged broiler chickens. The Vh, Cd, and the Vh/Cd are important indicators of intestinal digestion and absorption capacity, as an increase in Vh and Vh/Cd, and a shallowing of crypts indicate an improvement in gut nutrient digestion and absorption (Shirani et al., 2019). However, in most of these above-mentioned studies, the chickens were reared under stress conditions. It has been reported that prebiotics, including β-glucans and MOS, are most effective under disease and stress conditions, such as extremes of ambient temperature, crowding, and poor management or infectious diseases, which are invariably present in commercial broiler production (Fadl et al., 2020). The present study was conducted under good hygienic conditions (strict biosecurity measures, clean litter, good ventilation, and low stocking density), thus implying a minimum bacterial challenge. Under such conditions, the birds may not have required any functional feed to ameliorate their gut health. Furthermore, in the above-mentioned research, the birds received the functional feed until slaughtering age (35 d), while in the present study chickens only received the functional feed for 10 d, thus representing another potential factor that prevented the observation of any beneficial effects on gut morphometry. As a final aspect to consider, morphometric indices showed a proximo-distal decreasing gradient from the duodenum to the ileum in both the C- and the pYSM-fed birds, which is in accordance with the physiological processes of nutrient absorption in poultry (Murakami et al., 2007). Furthermore, feeding pYSM in prestarter and starter diets did not significantly influence the development or the severity of the histopathological changes detected in liver, gut or bursa of Fabricius of the chickens, thus suggesting that pYSM did not negatively affect gut health and animal health.

Feeding pYSM to broilers seemed also to drive the intestinal immune response toward an “anti-inflammatory pattern.” Indeed, it downregulated proinflammatory cytokines (IFN-γ, TNF-α, and IL-2) in the duodenum of broiler chicks in the prestarter phase (0–3 d). On the contrary, it did not influence the expression of anti-inflammatory IL-4. Inflammation plays a key role in protecting tissues after infection, but the uncontrolled inflammatory reaction characterized by a high release of proinflammatory cytokines would lead to tissue damage and high nutrient consumption (Klasing K.C., Science nutrition and the immune system, Br. Poult. Sci., 48, 2007, 525-537.). For this reason, the results obtained in the present study can be considered a positive outcome, helping in maintaining a proper balance of the intestinal cytokine levels and regulating the innate immune response. Very few studies are available on the effects of functional ingredients on gut inflammatory cytokines in poultry, especially in the prestarter and starter phases. However, it is well established that diet could be used to drive the intestinal immune response in poultry (Klasing K.C., Science nutrition and the immune system, Br. Poult. Sci., 48, 2007, 525-537.) and that β-glucans or MOS improved disease resistance against pathogens, enabling a low immune status and maximizing nutrients utilization for growth, rather than for the activation of the immune system in basal conditions (Agazzi et al., 2020). On the other side, in the case of bacterial infections, prebiotics improve the immune response, helping in controlling the disease (Teng and Kim, 2018). Indeed, Johnson et al. (2020) reported a decrease in proinflammatory cytokines after the administration of yeast β-glucans in chickens with necrotic enteritis, suggesting a controlled response situation. Furthermore, Janardhana et al. (2009) reported no significant differences in both the pro- and the anti-inflammatory cytokine transcription levels in chickens fed a functional feed containing MOS in basal conditions. Differently, Yitbarek et al. (2012) demonstrated that chickens receiving the same functional feed and infected with *Clostridium perfringens* presented high levels of proinflammatory cytokines (IL-12 and IFN-γ), supporting a proinflammatory effect via T-helper type-1 cell-associated pathways to control the early stages of the infection. These results are extremely heterogeneous, and they demonstrated that the interpretation of immune response is difficult because there is an active, homeostatic balance between proinflammatory and anti-inflammatory responses continuously occurring in the gut (Johnson et al., 2020). However, the results of the present study seem to suggest that β-glucans and MOS produce a low immune status in basal conditions in treated groups, even though further studies should be conducted in order to clarify the efficacy of such functional ingredient during a bird challenge.

On the contrary, MUC-2 transcription levels were similar in the duodenum of the C and T2 groups, but resulted to be lower in the T1 chickens (*P* = 0.016). Particularly, the MUC-2 gene encodes for secretory MUC-2, which is the primary gel-forming mucin in the gut (Zhang et al., 2015). Despite the statistical significance of the interaction between diet and gut segment, all the dietary treatments displayed normal levels of MUC-2 expression according to previous works (Hutsko et al., 2016; Ajuwon et al., 2020), and the differences recorded for the T1 group are still unclear. A possible explanation can be found in the different dosages of pYSM in the diets. In fact, Duangnumsawang et al. (2021) reported that thermal processing of poultry feed may reduce the mucus shedding in the lumen, reducing the stimulus to secrete mucins by goblet cells and, as a consequence, lowering the expression of the MUC-2 gene. The T1 group received a higher percentage of pYSM in their first 3 d of life, which could have had a protective role for the mucus layer, reducing the need of MUC-2 gene expression to replace it. Moreover, mucin transcription levels showed an increasing gradient from the duodenum to the jejunum. This is in accordance with the physiological development of mucin along the gut, and Forder et al. (2007) previously suggested that this can be due to an increase in bacterial colonization from the duodenum to the ileum that stimulates mucin production. Furthermore, the major effects observed in the duodenum of the treated groups may be due to the richness of the pYSM in highly digestible nutrients and fast digestible protein fraction. As a results, proteins reach their highest concentration in the duodenum, where they are rapidly absorbed. This can reasonably explain the immunomodulatory effect mainly seen in the duodenum, and, as a consequence, the lower effects in the jejunum, where the functional feed did not reach a sufficient concentration.

Regarding tight junctions, ZO-1 showed a statistical tendency at d 3, being lower in T1 compared to C and T2 groups (*P* = 0.085). Nonsignificant differences were observed for CL-1 in the birds at both d 3 and 10. Tight junctions, which seal the paracellular space between adjacent epithelial cells, are required for the maintenance of the mucosal barrier (Emami et al., 2019). Zona occludens-1 (**ZO-1**) localizes at the cytoplasmic surface of the cell membrane, close to the tight junction's strands, and it is thought to be a functionally critical tight junction component. Moreover, Claudin-1 (**CL-1**) is a pore-sealing claudin whose increased expression leads to a very tight epithelium, coinciding with an increased transepithelial electrical resistance and decreased solute permeability across the epithelial monolayer (Awad et al., 2017). Previous studies have demonstrated that dietary protein content and amino acids composition, along with probiotics and prebiotics administration, can improve tight junctions transcription levels in chickens challenged with *Eimeria/Salmonella* spp. or environmental stressors (e.g., heat stress) (Kitessa et al., 2014; Barekatain et al., 2019; Paraskeuas and Mountzouris, 2019; Santos et al., 2019). As already mentioned above, the statistical tendency observed for the lower expression of ZO-1 in T1 group could be due to the higher dosage of pYSM received by the birds during their first 3 d of life, which reduce the damage to the intestinal mucosa thanks to its lower content in ANFs (Duangnumsawang et al., 2021). However, the lack of more pronounced effects recorded in the present study on tight junctions could be attributed to the optimal conditions in which chickens were reared.

Independently of the age effect, feeding pYSM did not significantly influence the activities of sucrase and maltase enzymes. On the contrary, the aminopeptidase activity was clearly stimulated by pYSM utilization in broilers at either 3 or 10 d of age. An increased activity of disaccharidases (maltase and sucrase) and aminopeptidases in the small intestinal mucosa of broiler chickens has previously been reported as a result of an increased substrate presence at the apical membrane due to enhanced hydrolysis of dietary nutrients (Murugesan et al., 2014). Therefore, the so-obtained results suggests that pYSM utilization does not alter the carbohydrate availability in the small intestine, but is capable of influencing that of the proteins. This represents a reasonable outcome, as the product is a high digestible protein source. Furthermore, it is interesting to underline a more pronounced effect of high inclusion levels of pYSM (20%) in the prestarter feeding phase, while low inclusion levels (5%) seemed to be preferable in the starter period. This may reflect a time-related, dose-dependent response. Indeed, the T1-fed broilers with the 20% pYSM inclusion received a major amount of highly digestible nutrients in the first 3 d of life, thus probably determining a more pronounced digestion in this period and, in turn, potentially making the 10% pYSM inclusion level not effective enough in stimulating an analogous response (even if still similar to that underlined in the C group). On the contrary, the T2-fed broilers received the same amount of pYSM for all the 10 d of feeding, thus probably making the intestine needing a longer time to develop a more efficient enzymatic response (especially because the inclusion levels were low). Another aspect that is worthy to be highlighted is that the pYSM exerted a quite balanced effect on either the duodenum or the jejunum in terms of the aminopeptidase activity, even if a slightly more pronounced outcome was underlined in the duodenum in the starter feeding phase. This is in partial agreement with the down-regulation of the inflammatory cytokines and MUC-2 observed in the duodenum only. However, the higher enzymatic activities overall identified in the jejunum when compared to the duodenum (that were highlighted independently of the diet) reflect the role of the jejunum as primary site of nutrient digestion and absorption (Iji et al., 2001). Differently from the aminopeptidase modulation, feeding pYSM did not influence the activities of either the trypsin or chymotrypsin in broilers. This can still be considered a positive outcome, as an increase in such enzymes is commonly observed when ANFs are present in bird diets, thus representing a compensatory mechanism of pancreas (Rada et al., 2017; Erdaw et al., 2018). It is, however, interesting to underline that pYSM seemed to selectively stimulate specific proteases (aminopeptidase) rather than others (trypsin and chymotrypsin). Therefore, considering that each protease selectively catalyzes the hydrolysis of different amino acid sequences, the AA profile of the product—as well as the AA utilization by the bird microbiota—may have a key role.

In conclusion, the utilization of high dietary inclusion of a thermomechanical, enzyme-facilitated, coprocessed yeast and soybean meal in the first 10 d of life of the broiler chickens tended to improve bird growth performance in the prestarter and starter phases only, without negatively affecting organ weights, litter quality, leg health, and histopathological alterations. Despite the absence of pYSM-related effects on the gut morphological development and the activities of disaccharidases (sucrase and maltase) and pancreatic proteolytic enzymes (trypsin and chymotrypsin), feeding pSYM positively modulated the intestinal immune response (in terms of downregulation of proinflammatory cytokines) of birds during the first 3 d of life, as well as to stimulate the aminopeptidase activity in either the prestarter or the starter period. Further studies including pYSM in the entire production cycle of broilers, as well as assessing the pYSM-related modulation of the gut microbiota, are strongly recommended.

## DISCLOSURES

Two of the authors, Mark Karimi and Mai Anh Ton Nu, are employees of the AB NEO company. This interest has been fully disclosed to the journal.
